# Minimally Invasive Screw Fixation of Non-Pseudoarthorotic Lumbar Spondylolysis for Early Return to Sports

**DOI:** 10.7759/cureus.18323

**Published:** 2021-09-27

**Authors:** Hisanori Gamada, Masaki Tatsumura, Shun Okuwaki, Toru Funayama, Masashi Yamazaki

**Affiliations:** 1 Orthopedic Surgery, University of Tsukuba, Tsukuba, JPN; 2 Orthopaedic Surgery and Sports Medicine, Tsukuba University Hospital Mito Clinical Education and Training Center/Mito Kyodo General Hospital, Mito, JPN; 3 Orthopaedic Surgery, University of Tsukuba, Tsukuba, JPN

**Keywords:** buck’s method, early return to sports, cortical bone trajectory, minimally invasive surgery, lumbar spondylolysis

## Abstract

Lumbar spondylolysis is a fatigue fracture that occurs most frequently in middle and high school athletes. Conservative treatment is the first choice when the fracture is fresh. Surgical treatment of lumbar spondylolysis is often reported for pseudarthrosis cases, but surgery for cases of fresh fractures is rare. We report a case of a 16-year-old male, high jump athlete, with recurrent non-pseudoarthorotic lumbar spondylolysis.

He presented to our hospital with a chief complaint of back pain, and was diagnosed as right L5, pre-lysis-stage lumbar spondylolysis. After 3 months of conservative treatment, bone union was achieved with no obvious worsening of the fracture. His back pain also disappeared and he was able to return to exercise. At 6 months after the first examination, the lesion recurred and he could no longer continue playing sports, so surgical treatment was indicated.

Minimally invasive screw fixation was performed by combining Buck's method and the cortical bone trajectory. After the surgery, he started jogging at 5 weeks, resumed jumping practice at 7 weeks, and returned to competition at 2 months. He set a new personal best in a competition 3 months post-surgery. Bone union was achieved at 4 months. This technique is minimally invasive and does not involve debridement or bone grafting, which provides early pain relief and return to sports.

## Introduction

Lumbar spondylolysis is a fatigue fracture that most frequently occurs in middle and high school athletes. Conservative treatment is the first choice when the fracture is acute [1]. In cases of pseudarthrosis of the lesion, various surgical techniques have been described [2-5]. On the other hand, there are reports of surgery being performed in a very limited number of cases with acute fractures that do not respond to conservative treatment and are in persistent pain [6-7]. We report a case of recurrent non-pseudoarthorotic lumbar spondylolysis, in which a minimally invasive screw fixation was performed by combining Buck's method and the cortical bone trajectory (CBT) [8].

## Case presentation

**Figure 1:**
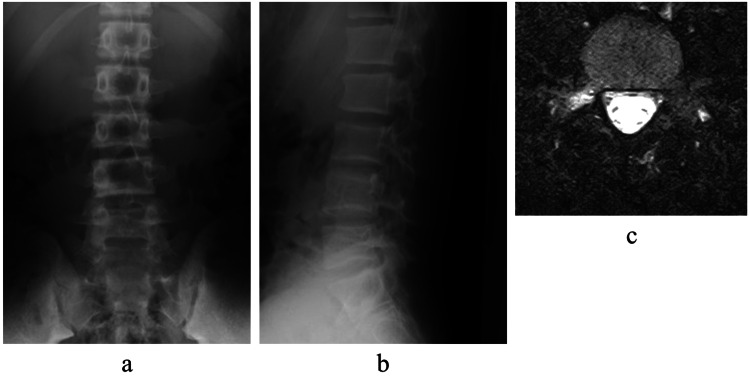
Plain radiographs and magnetic resonance images at first examination Plain radiographs at the first examination showed no obvious abnormalities (a, b). Short tau inversion recovery magnetic resonance images (STIR-MRI) showed high signal change in the right pedicle of L5 (c).

**Figure 2:**
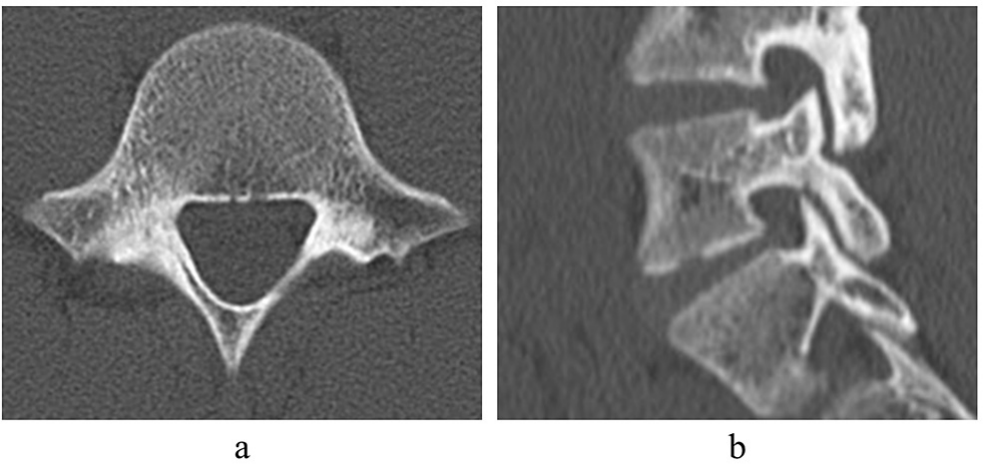
Computed tomography at first examination Computed tomography (CT) showed no fracture line in axial slice and slight bone resorption on the ventral side of the lamina in sagittal slice (a, b).

A 16-year-old high jump male athlete presented to our hospital with a chief complaint of back pain for two weeks. He was unable to continue practicing because his right back hurt during take-off. The pain was aggravated with back extension. Sciatic tension signs were negative and deep tendon reflexes in his lower limbs were normal. Plain radiographs showed no obvious abnormalities. Short tau inversion recovery magnetic resonance images (STIR-MRI) demonstrated a high signal change in the right pedicle of L5, and CT showed no fracture line in axial slice and slight bone resorption on the ventral side of the lamina in sagittal slice (Figures 1, 2).

**Figure 3:**
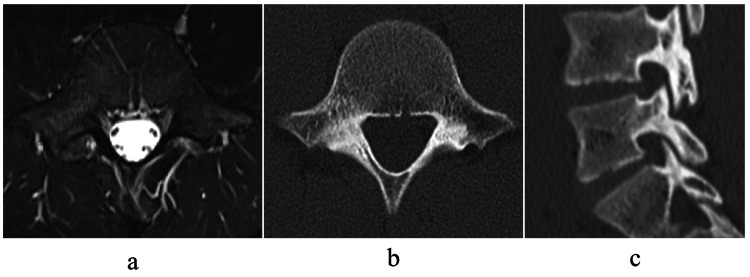
Imaging at 3 months after first examination Three months after the first examination, the signal change on magnetic resonance images (MRI) disappeared (a), and bone union was achieved with no obvious worsening of the fracture on computed tomography (CT) (b, c).

The patient was diagnosed as right L5, pre-lysis-stage lumbar spondylolysis. Conservative treatment such as wearing a hard Knight-type plastic brace, stopping training, and physical therapy was started [9]. After 3 months of conservative treatment, the signal change on MRI disappeared, and bone union was achieved with no obvious worsening of the fracture on CT (Figure 3).

**Figure 4:**
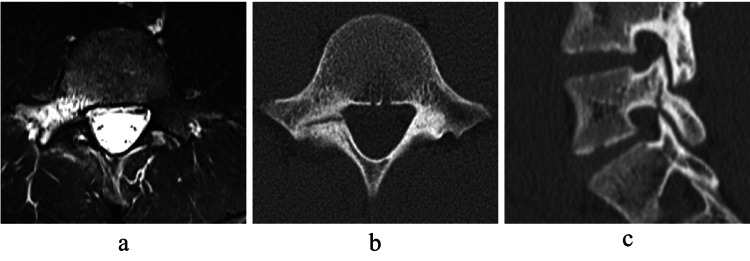
Imaging at 6 months after first examination Six months after the first examination, magnetic resonance images (MRI) showed high signal change (a), and computed tomography (CT) showed a hairline in axial slice (b) and an incomplete fracture in sagittal slice (c).

His back pain also disappeared and he was able to return to exercise. Six months past the first examination, back pain during exercise reappeared. Jumping was difficult due to the pain. MRI showed high signal change, and CT showed a hairline in the axial slice and an incomplete fracture in the sagittal slice. The patient was diagnosed with a recurrence of lumbar spondylolysis. The lesion was early stage and severer than that of the initial diagnosis (Figure 4). The initial conservative treatment took 3 months, but at the time of recurrence, the same or even longer treatment period may be required, and surgical treatment was considered indicated.

**Figure 5:**
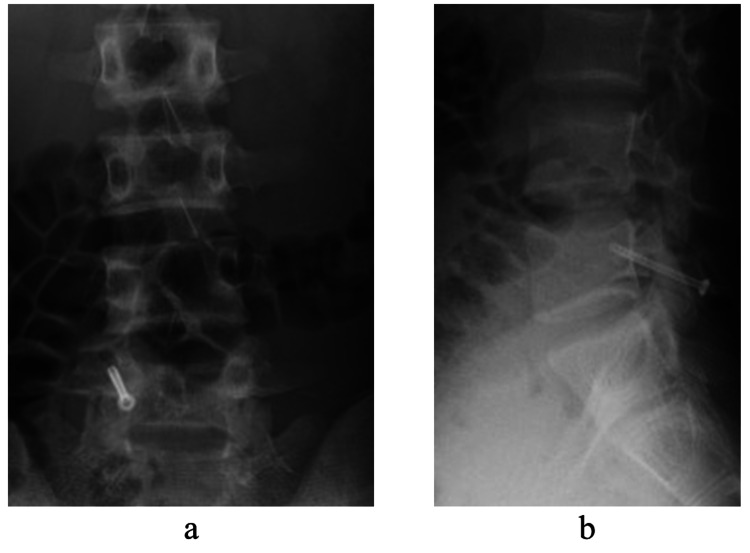
Postoperative plain radiographs Plain radiographs just after surgery (a, b).

**Figure 6:**
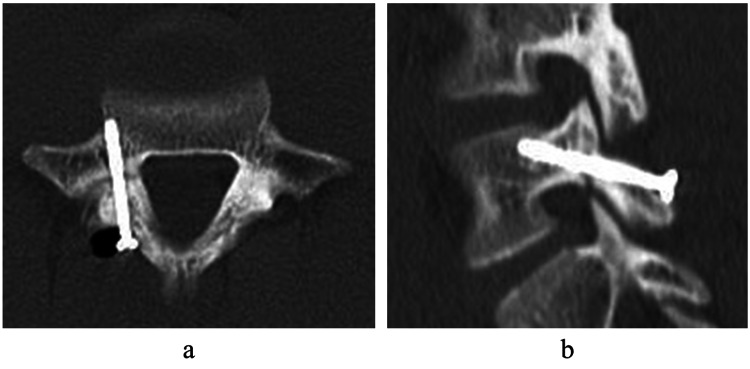
Postoperative computed tomography Postoperative computed tomography (CT) showed that the screw was inserted perpendicular to the fracture and in an appropriate position with sufficient contact with the surrounding cortical bone (a, b).

The surgery was performed under general anesthesia, in the prone position, and under endoscopy. The surgical instrument was a cannulated cancellous screw with a diameter of 4mm, a total length of 38mm, and a thread length of 16mm. A small skin incision was made on the L5 lamina, and a screw was inserted through the guide pin according to the CBT [8]. No debridement or bone graft of the lesion was performed. The postoperative plain radiograph and CT showed that the screws were inserted in the appropriate positions (Figures 5, 6).

**Figure 7:**
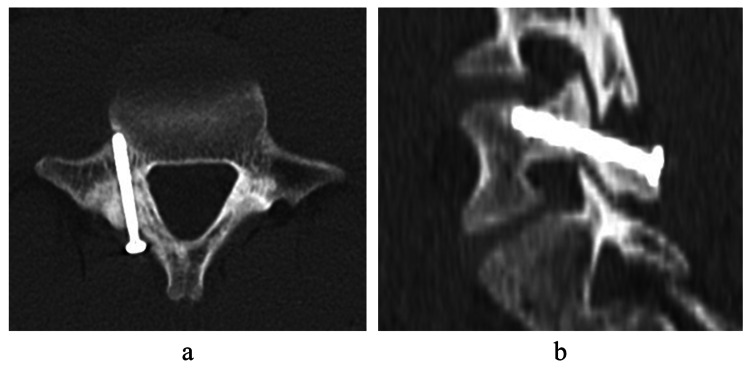
Postoperative radiographs at 4 months Computed tomography (CT) images 4 months after surgery. The lesion achieved bone union (a, b).

The patient started walking with a corset on the day after the surgery. After the pain at the surgical site and the former back pain disappeared, he started jogging at 5 weeks, resumed jumping practice at 7 weeks, and returned to competition at 2 months. He set a new personal best in a competition 3 months after his surgery. Four months after the surgery, bone union was confirmed by CT (Figure 7). He is currently undergoing outpatient follow-up with no back pain. As of 6 months post-surgery, he continues to compete without back pain and is being followed up.

## Discussion

In the treatment of lumbar spondylolysis, there are many reports on conservative treatment for early return to sports and the use of low-intensity pulsed ultrasound to improve the rate of bone union [10-11]. However, the rate of bone union with conservative treatment is 76-94%, and there is still a risk of prolonged pain if bone union is not achieved [9,12-13].

For fractures of other parts of the body, there are early surgical options such as screw fixation for fractures that are expected to have poor bone union rates with conservative treatment, such as scaphoid fractures of the hand [14]. In addition, "Jones fracture," a fatigue fracture of the base of the fifth metatarsal, which is a common occurrence in athletes, is often treated with screw fixation for athletes who wish to return to sports as soon as possible, as bone healing is likely to be prolonged even if the fracture is incomplete [15].

Various surgical methods have been reported for lumbar spondylolysis, including the Buck method, the Scott method, pedicle screw and segmental wire fixation, pedicle screw-rod-hook fixation, and recently, the Smiley face rod method. All of these methods have been reported to be effective in treating pseudarthrosis [2-5]. Among them, there are various reports of screw fixation, including robot assistance and navigation. In both reports, surgery was performed for pseudoarthorotic lesions after 6 to 60 months of conservative treatment, with debridement of the lesion or bone graft [2,16-18]. However, even in fresh lesions without pseudarthrosis, surgery may be indicated in cases that are resistant to conservative treatment and persistent in pain [6-7].

In this study, we performed Minimally invasive screw fixation using Buck's method applied as osteosynthesis in a patient with a recurrent, non-pseudoarthorotic, early, unilateral lesion. The initial conservative treatment took three months. When the lesion recurred, a longer treatment period was expected. Therefore, minimally invasive surgery was indicated for early return to sports. One of the innovations of this technique, which is based on the Buck method, is the use of CBT for screw insertion [8]. For the purpose of osteosynthesis, sufficient initial fixation is required both proximal and distal to the lesion. The screw was inserted using the trajectory of the CBT [8]. Since the correct CBT is not easy to create the entry point, the endoscopic assist was useful in creating the appropriate entry point while minimizing muscle injury [19]. Because the lesion, in this case, is a fresh fracture with bone marrow edema and not a pseudoarthrosis even though there is a gap in the fracture, we believe that debridement of the lesion or bone graft, which are performed in conventional screw fixation, are unnecessary [2, 16-18]. This technique is minimally invasive, with a small skin incision, almost no soft tissue dissection, no debridement of the lesion, and no bone grafting. Early pain relief was achieved due to the fixation of the screw. He was also able to resume exercise about a month post-surgery and return to competition within two months. Bone union was achieved in four months. Although this case was a recurrence after conservative treatment, we believe that internal fixation will eliminate the risk of recurrence and allow the patient to compete without worrying about recurrence in the future. In addition, it has been reported that the results of conservative treatment for contralateral pseudoarthrosis in bilateral lesions are very poor [20]. Therefore, we believe that internal fixation of the unilateral lesion to ensure bone union may stop the negative cycle of fractures and pseudoarthrosis.

On the other hand, there are some issues to be addressed. The long-term outcome of this procedure is still unknown. Since this is a surgery for adolescent patients, long-term follow-up and research with more cases are essential. It is also a question of what stage of the lesion can be treated with this technique. Since this technique can apply a compression force to the lesion but does not involve debridement of the lesion, we consider that it is indicated for pre-lysis to progressive lesions with bone marrow edema.

In our opinion, this technique is suitable for patients who are unable to return to sports due to pain, patients with pre-lysis to progressive lesions with bone marrow edema, patients with prolonged treatment and sports interruption such as recurrent cases, and top athletes who wish to return to sports as soon as possible.

## Conclusions

Minimally invasive screw fixation combined with Buck's method and CBT was performed for a patient with recurrent non-pseudoarthorotic lumbar spondylolysis. This technique provided early pain relief and the patient was able to return to sports early. We believe that this technique will be helpful in the treatment of similar cases.
